# Efficacy and Affecting Factors of ^131^I Thyroid Remnant Ablation After Surgical Treatment of Differentiated Thyroid Carcinoma

**DOI:** 10.3389/fonc.2018.00640

**Published:** 2018-12-20

**Authors:** Chen Wang, Hongcui Diao, Ping Ren, Xufu Wang, Yangang Wang, Wenjuan Zhao

**Affiliations:** ^1^Department of Endocrine and Metabolic Diseases, the Affiliated Hospital of Qingdao University, Qingdao, China; ^2^Department of Endocrine, Yiyuan County People's Hospital, Zibo, China; ^3^Department of Nuclear Medicine, the Affiliated Hospital of Qingdao University, Qingdao, China

**Keywords:** differentiated thyroid carcinoma, iodine-131, remnant ablation, thyroglobulin, affecting factors

## Abstract

**Purpose:** Radioiodine (^131^I) thyroid remnant ablation is an important treatment of differentiated thyroid carcinoma (DTC) and various factors affecting its efficacy have been reported but not well defined. The aim of our study was to evaluate the efficacy and the affecting factors of ^131^I ablation after total or near-total thyroidectomy in a relative large DTC cohort.

**Methods:** 261 DTC patients with negative thyroglobulin antibody received 100–200 mCi ^131^I for thyroid remnant ablation after total or near-total thyroidectomy between January 2012 and October 2015 in our hospital. The efficacy and affecting factors of ^131^I ablation therapy were retrospectively investigated.

**Results:** The success rate of the first ^131^I thyroid remnant ablation was 65.90%. Univariate analysis demonstrated that larger tumor size, higher level of pre-ablation stimulated thyroglobulin (sTg), intermediate to high risk stratification for recurrence, and lymph node and distant metastases were associated with a lower success rate of the first ^131^I ablation (all *p* < 0.05). Multivariate logistic regression analysis showed that tumor size, pre-ablation sTg, and lymph node and distant metastases were independent factors affecting the efficacy of the first ^131^I ablation. Areas under receiver operating characteristic curves for sTg, sTg/TSH ratio, and tumor size to predict unsuccessful ablation were 0.831, 0.824, and 0.648, respectively. The threshold values were 4.595 ng/ml, 0.046 mg/IU, and 1.350 cm, respectively. The sensitivities were 95.51, 96.63, and 73.03% and the specificities were 64.54, 61.63, and 49.41%, respectively. The excellent response (ER) ratio of the successful group was significantly higher than that of the unsuccessful group.

**Conclusions:** The efficacy of the first ^131^I thyroid remnant ablation after surgical treatment of DTC is well demonstrated, and tumor size, pre-ablation sTg, lymph node, and distant metastases are independent factors affecting its efficacy.

## Introduction

Thyroid carcinoma is the most common endocrine malignant tumor and its incidence is on the rise worldwide (1). Differentiated thyroid carcinoma (DTC) accounts for 90% of all thyroid cancers, including papillary thyroid carcinoma (PTC) and follicular thyroid carcinoma (FTC) (2). DTC patients usually have a good prognosis, but cervical lymph node metastasis and distant organ metastasis are frequently found, which may have a negative impact on the prognosis of DTC patients. The overall recurrence and mortality rates for DTC are 20.5 and 8.5%, respectively, over a mean follow-up period of 11.3 years (3). Patients with neither distant metastasis nor recurrence within 3 years after primary surgery have a 10-year survival rate of 96%, whereas patients without distant metastasis but with recurrence within the first 3 years have a 10-year survival rate of 48% (4). However, patients with distant metastasis identified within 3 years after surgery have a 10-year survival rate of 33% (4). Standard treatments for DTC include surgery, selective radioactive iodine-131 treatment, and thyroid stimulating hormone (TSH) suppression therapy. ^131^I thyroid remnant ablation, which is an important treatment for DTC, plays a key role in eliminating residual thyroid tissue and potential microscopic residual tumor foci after total or near-total thyroidectomy. Primary site cancer recurrence, distant metastasis recurrence, and disease-specific patient death rates are significantly lower after ^131^I thyroid remnant ablation than those with TSH suppression alone or no postoperative medical therapy (5). The efficacy of ^131^I thyroid remnant ablation can be varied significantly among DTC patients with different characteristics, such as surgery type, size of residual thyroid tissue, tumor size, and TSH level. Moreover, the efficacy of ^131^I thyroid remnant ablation varies considerably across published studies, and the affecting factors are still not well defined. The aim of this study was to evaluate the efficacy and the affecting factors of the first ^131^I thyroid remnant ablation after total or near-total thyroidectomy in DTC patients.

## Materials and Methods

### DTC Patients

In this retrospective study, 304 consecutive patients with DTC received ^131^I thyroid remnant ablation after total or near-total thyroidectomy between January 2012 and October 2015 at the Nuclear Medicine Department in Affiliated Hospital of Qingdao University, China. Inclusion criteria were as follows: (1) patients with histological confirmation of DTC, (2) patients who received total or near-total thyroidectomy, and (3) patients who met the treatment criteria of ^131^I thyroid remnant ablation and received ^131^I ablation. Exclusion criteria were as follows: (1) patients with positive thyroglobulin antibody, (2) patients whose TSH level was <30 mIU/L before ^131^I ablation, and (3) patients who lost to follow-up and had inadequate information. Near-total thyroidectomy was as classically defined; i.e., the estimated residual thyroid tissue after thyroidectomy was <1.0 g. Total or near-total thyroidectomy was treated equally in our study, which is consistent with the recommendations of American Thyroid Association guidelines as well as Chinese Thyroid Association guidelines on the management of DTC (6, 7). Therefore, in the present study, patients with near-total thyroidectomy and also received ^131^I thyroid remnant ablation were included. Of the 304 potential study subjects, 43 patients were excluded from the study, among whom 2 had positive serum thyroglobulin antibody (TgAB) and 41 lost follow-up with inadequate information. None of the patient with TSH <30 mUI/L was included in the study. Therefore, 261 patients were finally enrolled in the study. The TgAb was negative in the DTC patients analyzed. The detection range of TSH and sTg were 0.005–100.00 mIU/L and 0.04–1000.00 ng/ml, using Electrochemiluminescence immunoassay (ECLIA, Roche Germany). We collected the following information: surgery type, histological type, with or without Hashimoto thyroiditis (HT), unilateral or bilateral foci, number of foci, TNM stage, dosage of ^131^I, time interval between surgery and the first ^131^I ablation, tumor size, risk stratification for recurrence (7), lymph node metastasis, distant metastasis, TSH measurement, and sTg before ^131^I ablation.

### ^131^I Thyroid Remnant Ablation

According to the 2009 ATA guideline and 2012 Chinese guideline (6, 7), the following are indications for ^131^I treatment: (1) DTC with lymph node metastasis or distant metastasis after thyroidectomy, highly invasive histological features such as tall cell variant, columnar cell variant, hobnail variant and papillary carcinoma with a focal undifferentiated component, and extrathyroidal extension; (2) tumor size >4 cm even in the absence of other higher risk features; and (3) for the patients having tumor size <4 cm with preoperative lymph node metastasis but absence of postoperative metastasis, the treatment decision will be made based on the physician's judgment and the patient's wishes. If the tumor diameter, preoperative lymph node status (site and number), and age are indicative for the increased risk of the future recurrence and metastasis, ^131^I ablation therapy is recommended. ^131^I treatment is not recommended for all low-risk DTCs with tumor lesions <1 cm and no extra-glandular infiltration, no lymph nodes, and absence of distant metastasis.

All patients avoided taking levothyroxine (L-T4) after thyroid operation or stopped taking L-T4 for 3–4 weeks to raise the endogenous TSH level>30 mIU/L before ^131^I ablation. To achieve urinary iodine <100 ug/L before ^131^I ablation, all patients were required to maintain a low-iodine diet (<50 ug per day) for 1–2 weeks and to avoid drugs containing iodine as well as contrast agent. Following tests were completed within 1 week before ^131^I ablation, including TSH, free thyroxine, free triiodothyronine, thyroglobulin, TgAb, neck ultrasound, and chest CT scan to rule out contraindications for ^131^I ablation and to evaluate the size of residual thyroid tissue and the presence of pulmonary metastasis. On the day immediate before ^131^I ablation treatment, 99mTcO-4 thyroid static imaging and urine iodine determination were performed to assess the size of residual thyroid and the level of urine iodine for helping determination of the dose of ^131^I. Treatment dose of ^131^I (atomic hi-tech co., LTD) was given on an empty stomach. Subjects were routinely given a dose of 100 mCi (3.7 GBq, 1 mCi = 37 MBq) except for patients with suspected or confirmed microscopic residual lesions, lymph node metastasis, distant metastasis or unexplained serum Tg level elevation who were given a higher dose of ^131^I (100–200 mCi) according to the disease extent (e.g., extent of metastasis and the degree of rising Tg level).

### Evaluation of Therapeutic Effect of ^131^I Thyroid Remnant Ablation

Approximately 6 months after ^131^I ablation, treatment effect was evaluated. ^131^I thyroid remnant ablation was considered to be successful when subjects met the following two conditions, as previously reported (8, 9): (1) serum stimulated thyroglobulin was <1 ng/ml or suppressed thyroglobulin was <0.2 ng/ml and (2) absence radioactive iodine uptake in the thyroid bed according to a diagnostic iodine-131 whole-body scan.

### Clinical Efficacy Evaluation

The risk assessment after the initial ^131^I treatment was analyzed according to the 2015 ATA guideline (8) and the clinical therapeutic results was divided into 4 categories: the satisfactory curative effect (Excellent Response, ER), the uncertainty effect (Indeterminate Response, IDR), poor biochemical effect (Biochemical Incomplete Response, BIR), and poor structural effect (Structural incomplete Response, SIR).

### Statistical Analyses

Statistical analysis was performed using the SPSS 17.0 statistical software. The data were expressed as mean ± standard error (S.E.) or median. Student-*t* test was used to examine the difference in the means between groups. Categorical data was presented as percentage and comparisons between groups were analyzed by chi-square test. Affecting factors to the efficacy of ^131^I ablation were assessed by analyzing the differences of clinical and laboratory parameters between the successful and unsuccessful ablation groups. To identify independent affecting factors of the efficacy of ^131^I ablation, multivariate logistic regression analysis was performed by adjusting for possible confounding factors. Receiver operating characteristic (ROC) curve analysis was constructed to determine the best cut-off values of sTg, sTg/TSH ratio and tumor diameter before ^131^I ablation for predicting the unsuccessful ablation and to assess the predict values of the affecting factors. Correlation analysis was performed via Spearman bivariate correlation analysis. A *p* < 0.05 was considered as statistically significant.

## Results

### The Efficacy of the First ^131^I Thyroid Remnant Ablation

Of the total of 261 DTC patients receiving ^131^I thyroid remnant ablation, the first ^131^I thyroid remnant ablation succeeded in 172 patients and failed in the remaining 89 patients, with a success rate of 65.90%.

### Clinical Evaluation of Therapeutic ^131^I Thyroid Remnant Ablation

Two-Hundred and Sixty-one patients were finally enrolled for analysis, including 80 males and 181 females, aged 8–79 years, with an average age of 44 years. Among them, 9 cases were ≤18 years old (children and adolescents), 3 cases were successfully ablated (3/9, 33.33%); and 252 cases were >18 years old (adult), 169 cases were successfully ablated (169/252, 67.06%), there was no significant difference in the success rate of ablation between the two groups (*X*^2^ = 1.700, *p* = 0.182). Accordingly, there was no significant difference of the successful ablation between the patients <45 years old and those ≥ 45 years old (Table 1). The total thyroidectomy was performed in 203 cases, including lymph node dissection in 191 cases and the subtotal thyroidectomy was operated in 58 cases, including lymph node dissection in 30 cases. Two Hundred and Thirty-Five cases were papillary thyroid carcinoma and 26 cases follicular carcinoma, with 24 cases combined with Hashimoto's disease. The numbers of the primary carcinomas were 1–7. The range of TSH before ^131^I ablation was 32–100 mIU/L. The time range between the first ^131^I ablation and surgery were 3 weeks to 23 years. According to the pathological results, the size of primary carcinomas (diameter) was 0.1 cm to 6 cm, with a median of 1.5 cm. The minimum, maximum, and median tumor sizes of the successful and unsuccessful ablation groups were 0.1, 5.0, 1.5 cm and 0.3, 6.0, 1.7 cm, respectively. The size of primary carcinomas in the successful group was smaller than that in the unsuccessful group, and the difference was statistically significant (*p* < 0.05) (Table 2). The serum thyroglobulin (sTg) level ranged 0.04–1000 ng/ml, and the sTg/TSH level was 0.0004–24.5633 mg/IU. Age, gender, surgery type, histological type, concurrent HT, number of foci, unilateral or bilateral foci, TSH level, time interval between surgery and first ^131^I ablation, and TNM stage displayed no significant differences between the successful and unsuccessful ablation groups (all *p* > 0.05) (Table 1). In contrast, tumor size, pre-ablation sTg, sTg/TSH ratio, dose of ^131^I, risk stratification for recurrence, lymph node metastasis, and distant metastasis showed significant differences between the successful and unsuccessful ablation groups (all *p* < 0.05). Tumor diameter > 1.680 cm, pre-ablation sTg > 5.450 ng/ml, sTg/TSH ratio > 0.066 mg/IU, higher dose of ^131^I, intermediate- to high-risk stratification for recurrence, and lymph node metastasis and distant metastasis were significantly associated with unsuccessful ablation (Table 3).

**Table 1:** Comparison of the efficacy of the first ^131^I ablation between various affecting factors (*n*, %).

**Index**	**N**	**Successful ablation [*n* (%)]**	***X*^**2**^**	***p***
Age (Year)			0.363	0.547
<45	134	86 (64.18)		
≥45	127	86 (67.72)		
Gender			2.625	0.105
Male	80	47 (58.75)		
Female	181	125 (69.06)		
Operation method			1.759	0.186
Total thyroidectomy	203	138 (67.98)		
Near-total thyroidectomy	58	34 (58.62)		
Histological type^*^			0.003	0.953
PTC	235	155 (65.96)		
FTC	26	17 (65.38)		
HT		0.007	0.934	
Yes	24	16 (66.67)		
No	237	156 (65.82)		
Number of foci			3.009	0.390
1	120	85 (70.83)		
2	91	55 (60.44)		
3	28	19 (67.86)		
≥4	22	13 (59.09)		
Unilateral or bilateral foci			2.314	0.128
Unilateral	166	115 (69.28)		
Bilateral	95	57 (60.00)		
TSH (mIU/L)			0.265	0.876
30–59	30	16 (53.33)		
60–89	51	26 (50.98)		
≥90	180	99 (55.00)		
Time interval between surgery and first ^131^I ablation (Month)			1.586	0.811
≤1	93	64 (68.82)		
1~2	112	74 (66.07)		
2~3	25	15 (60.00)		
3~6	18	12 (66.67)		
>6	13	7 (53.85)		
TNM stage			0.155	0.693
I-II	151	101 (66.89)		
III-IV	110	71 (64.55)		

* *Postoperative pathological diagnosis; PTC, papillary thyroid carcinoma; FTC, follicular thyroid carcinoma*.

**Table 2:** Comparison of tumor diameters between the first ^131^I ablation successful group and the unsuccessful group (cm).

**Index**	**Minimum**	**Maximum**	**Median**	**Average**	**S. E**.
Successful group	0.1	5.0	1.5	1.55	0.08
Unsuccessful group	0.3	6.0	1.7	1.95	0.12
Total	0.1	6.0	1.5	1.68	0.07

*T = 2.941, p = 0.004. S. E, standard error*.

**Table 3:** Comparison of the efficacy of the first ^131^I ablation between groups with different clinical and laboratory parameters (*n*, %).

**Index**	**Number (*n*)**	**Successful ablation [*n* (%)]**	**X^**2**^**	***p***
Tumor diameter (cm)			11.418	0.001
≤1.68^#^	149	111 (74.50)		
>1.68	112	61 (54.46)		
sTg (ng/ml)		72.795	0.000	
≤5.45^※^	131	119 (90.84)		
>5.45	130	53 (40.77)		
sTg/TSH (mg/IU)		68.407	0.000	
≤0.066^※^	131	118 (90.08)		
>0.066	130	54 (41.54)		
DOSE of ^131^I (mCi)		20.215	0.000	
100	168	127 (75.60)	7.165^a^	0.007^a^
120	34	18 (52.94)	17.812^b^	0.000b
150–200	59	27 (45.76)	0.445^c^	0.505^c^
Risk stratification of recurrence			8.721	0.013
Low risk	24	22 (91.67)	6.127^d^	0.013^d^
Intermediate risk	113	75 (66.37)	8.657^e^	0.003^e^
High risk	124	75 (60.48)	0.882^f^	0.348^f^
With or without metastasis			24.661	0.000
No metastasis^*^	40	37 (92.50)	8.823^g^	0.003^g^
No postoperative metastasis^**^	133	92 (69.17)	19.090^h^	0.000^h^
Postoperative lymph node metastasis^***^	38	18 (47.37)	18.728^i^	0.000^i^
Distant metastasis^****^	50	25 (50.00)	6.124^j^	0.013^j^
			5.793^k^	0.016^k^
			0.060^l^	0.807^l^

# 1.68 cm is the mean of the tumor size;

※ 5.45 ng/ml is the median of sTg; 0.066 mg/IU is the median of sTg/TSH ratio;

* There was no metastasis before and after surgery;

** Surgery had the lymph node metastasis cleaned and no lymph node and distant metastasis was found after surgery;

*** There were still lymph node metastasizes but no distant metastasis after surgery;

**** All of the patients had lymph node metastasis, among them 39 cases of lung metastasis, 6 cases of bone metastasis, 5 cases of both bone and lung metastasis.

a, b *100mCi group compared with 120mCi, 150~200mCi groups*.

c *120mCi group compared with 150~200mCi group*.

d, e *low risk group compared with intermediate and high risk groups, respectively*.

f *intermediate risk group compared with high risk group*.

g−i *no metastasis group compared with without postoperative metastasis, postoperative lymph node metastasis and distant metastasis groups, respectively*.

j, k *without postoperative metastasis group compared with postoperative lymph node metastasis and distant metastasis groups*.

l *postoperative lymph node metastasis group compared with distant metastasis group*.

### Independent Affecting Factors of the Efficacy of ^131^I Thyroid Remnant Ablation

Multivariate logistic regression analysis showed that tumor size, pre-ablation sTg, lymph node metastasis and distant metastasis were the independent factors affecting the efficacy of ^131^I thyroid remnant ablation in DTC patients (Table 4). However, intermediate- to high-risk stratification for recurrence was not a factor independently affecting the efficacy of ^131^I thyroid remnant ablation in DTC patients (Table 4) because such risk stratification itself contained multi risk factors.

**Table 4:** Multivariate logistic regression analyses to identify independent affecting factors of the efficacy of ^131^I ablation.

	**B**	**S.E**.	**Wals**	**df**	**Sig**.	**Exp (B)**
Tumor diameter	−0.465	0.138	11.301	1	0.001	0.628
sTg	−0.002	0.001	8.856	1	0.003	0.998
Metastasis situation			10.900	3	0.012	
No metastasis after operation	−1.000	0.851	1.383	1	0.240	0.368
Lymph node metastasis after operation	−2.244	0.783	8.217	1	0.004	0.106
Distant metastasis	−1.585	0.748	4.491	1	0.034	0.205
Intermediate-to high-risk	−0.346	0.917	0.142	1	0.706	0.708
Constant	3.494	0.830	17.741	1	0.000	32.928

*B, partial regression coefficient; S.E, standard error; Wals, Wald statistics; EXP (B), the OR value of the corresponding variable*.

### The ROC Curves for sTg, sTg/TSH Ratio and Tumor Size Before ^131^I Ablation to Predict Unsuccessful Ablation

**Figure 1:**
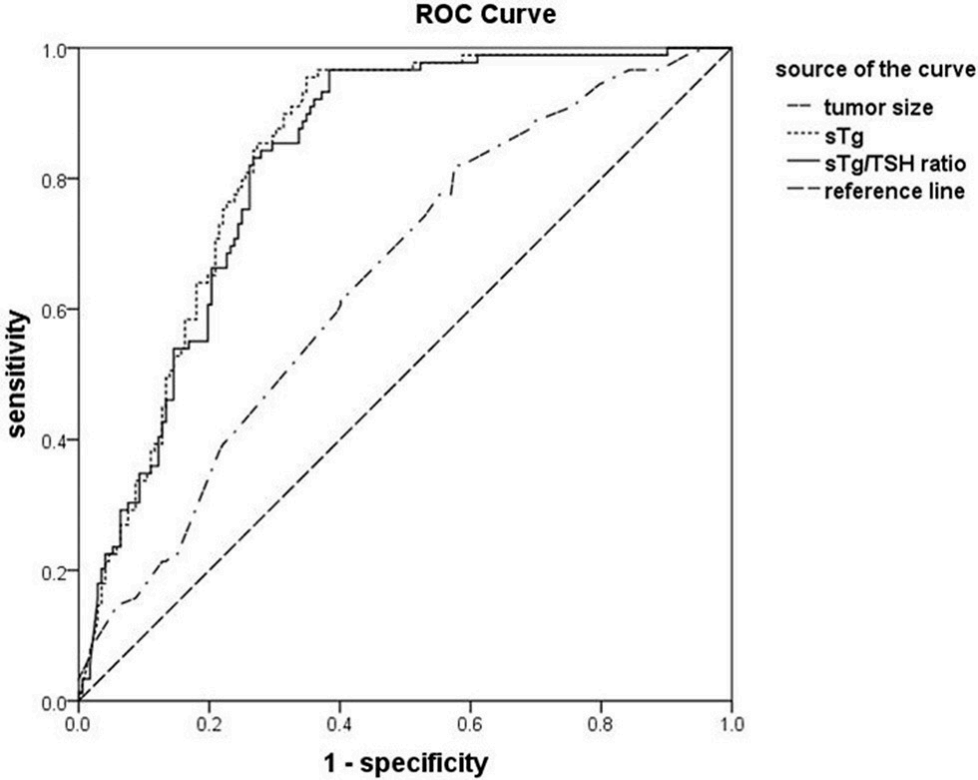
The receiver operating characteristic (ROC) curves for sTg, sTg/TSH ratio and tumor size before ^131^I ablation to predict unsuccessful ablation. ROC curve analysis was constructed to determine the best cut-off values of sTg, sTg/TSH ratio, and tumor diameter (as indicated) before ^131^I ablation for predicting the unsuccessful ablation and to assess the predict values of the affecting factors.

The areas under ROC curve for sTg, sTg/TSH ratio and tumor size to predict unsuccessful ablation were 0.831, 0.824, and 0.648, respectively. The threshold values were 4.595 ng/ml, 0.046 mg/IU, and 1.350 cm, respectively. The sensitivities were 95.51, 96.63, and 73.03%, respectively, and the specificities were 64.54, 61.63, and 49.41, respectively. The negative predictive values were 96.52, 97.25, and 77.98%, respectively (Figure 1).

### Efficacy Evaluation Among Different Metastatic Groups After ^131^I Ablation

The clinical efficacy of ^13^^1^I ablation after treatment was evaluated and compared in four groups: (1) no metastasis (absence of metastasis before and after surgery), (2) no postoperative metastasis (surgery had the metastatic lymph nodes cleaned. No lymph node and distant metastasis was found after surgery), (3) postoperative lymph node metastasis (metastatic lymph nodes were found but no distant metastasis after surgery), and (4) distant metastasis (all patients had lymph node metastases concurrently with distant metastases; including 39 cases of lung metastasis, 6 cases of bone metastasis, and 5 cases of both bone and lung metastasis). Results showed that the difference between 4 groups was statistically significant (*p* = 0.000), and the pairwise comparison between the other three groups was statistically significant (all *p* < 0.008) except for non-metastasis group and postoperative non-metastasis group (*p* > 0.008). The ER rates in the non-metastasis group, the postoperative non-metastasis group, the postoperative lymph node metastasis group, and the distant metastasis group decreased gradually, while the SIR rate showed an upward trend, and no significant regularity were found for the IDR rate and BIR rates. Further comparison of the ER rates between the groups showed that the non-metastasis group and the postoperative non-metastasis group were higher than those in the postoperative lymph nodes metastatic group or distant metastasis group (both *p* < 0.008); postoperative lymph node metastasis group ER rate was higher than that of distant metastasis group (*p* < 0.008), except that there was no significant difference between the non-metastasis group and the postoperative non-metastasis group (*p* > 0.008, Table 5). The correlation analysis showed that ER rate was negatively correlated with metastasis (*r* = −0.99, *p* = 0.0096). Then we analyzed the success rate of ^131^I and ER rate, the ER ratio of the successful group was 74.42% (128/172), and the ER ratio of the unsuccessful group was 5.62% (5/89), the difference was statistically significant (*X*^2^ = 111.092, *p* = 0.000).

**Table 5:** Comparison of clinical efficacy between different metastatic groups 6 months after the first ^131^I ablation (*n*, %).

**Index**	**N**	**ER**	**IDR**	**BIR**	**SIR**	**No ER^※^**
No metastasis^*^	40	34 (85.00)	6 (15.00)	0 (0.00)	0 (0.00)	6 (15.00)
No postoperative metastasis^**^	133	89 (66.92)	33 (24.81)	11 (8.27)	0 (0.00)	44 (33.08)
Postoperative lymph node metastasis^***^	38	10 (26.32)	9 (23.68)	5 (13.16)	14 (36.84)	28 (73.68)
Distant metastasis^****^	50	0 (0.00)	0 (0.00)	0 (0.00)	50 (100.00)	50 (100.00)
Total	261	133 (50.96)	48 (18.39)	16 (6.13)	64 (24.52)	128 (49.04)
X^2^^#^ = 228.262, 4.066^a^, 32.661^b^, 90.000^c^, 60.290^d^, 177.000^e^, 43.421^f^						
*p* = 0.000, 0.131^a^, 0.000^b^, 0.000^c^, 0.000^d^, 0.000^e^, 0.000^f^						
X^2^^##^ = 93.290, 4.893^g^, 27.293^h^, 68.304^i^, 19.987^j^, 65.138^k^, 14.845^l^						
*p* = 0.000, 0.019^g^, 0.000^h^, 0.000^i^, 0.000^j^, 0.000^k^, 0.000^l^						
*r* = −0.99 *P* = 0.0096						

※ denotes IDR, BIR, SIR;

# the comparison of clinical effect;

## *the comparison of ER rate*.

a, b, c and

g, h, i indicate no metastasis group compared with no postoperative metastasis, postoperative lymph node metastasis and distant metastasis groups, respectively;

d, e and

j, k no postoperative metastasis group compared with postoperative lymph node metastasis group and distant metastasis group;

f, l *postoperative lymph node metastasis group compared with distant metastasis group*.

* There was no metastasis before and after surgery;

** Surgery had the lymph node metastasis cleaned and no lymph node and distant metastasis was found after surgery;

*** There were still lymph node metastasizes but no distant metastasis after surgery;

**** *All of the patients had lymph node metastasis, among them 39 cases of lung metastasis, 6 cases of bone metastasis, 5 cases of both bone and lung metastasis*.

## Discussion

Although DTC is a tumor with a high degree of differentiation, disease recurrence or metastasis in DTC patients after surgery remains approximately 10–30% (10, 11). ^131^I therapy, which is a major adjuvant treatment for DTC after thyroid surgery, can reduce risk of recurrence or metastasis and improve the survival of patients with DTC (12).

The success rate of the first ^131^I ablation after surgical treatment of DTC varies in the published literature, and its affecting factors are not well defined. Previous studies report that the success rates of the first ^131^I thyroid remnant ablation range from 43 to 87.2% (13–16), and some factors could possibly affect the therapeutic effectiveness, such as patient gender, surgery type, size of residual thyroid tissue, tumor size, TSH level, and thyroglobulin level (17–19). Considering the age range of this study population (8–79 years), to rule out the effect of age difference on the success rate of ^131^I ablation, we compare the success rates between the aged ≤18 years (children and adolescents) group and >18 years old (adults) group and between the group aged <45 years old and ≥45 years old group. Results show no significant difference, indicating the heterogeneity caused by age stratification is negligible. This is consistent with the ATA guideline (7) that recommends the treatment of adults apply equally to children and adolescents. The first ^131^I ablation success rate of 261 DTC patients enrolled in our retrospective analysis is 65.90%, within the range of published data (13–16). Additionally, our data show that tumor size, pre-ablation sTg, risk stratification for recurrence, lymph node metastasis and distant metastasis are the major determinants affecting the clinical efficacy of ^131^I ablation therapy. The other factors, however, such as age, gender, histological type, and TNM stage, had no significant effects on ^131^I ablation therapy.

Many studies report that, when the TSH level is greater than or equal to 30 mIU/L, the success rate of ^131^I ablation often reach to maximum, but further higher TSH level does not improve the success rate of ^131^I ablation in parallel (15, 18, 20). Accordingly, current established guidelines recommend that, for the ^131^I ablation therapy to be effective, the preferred serum TSH level before ^131^I ablation should be higher than or equal to 30 mIU/L (6, 9) and the side effects caused by higher TSH level can also be minimized. In this study, no statistically significant difference is observed for the success rate of the first ^131^I ablation between the groups with different TSH level. Fu et al. (20) report similar findings. In contrast, Zhao et al. (21) find that patients with low- to intermediate-risk DTC respond better to ^131^I therapy when the pre-ablative TSH level is 90 to <120 μIU/ml. These inconsistent results may be explained by the different inclusion criteria or different baseline characteristics of the DTC patients included in the different studies.

The dose of ^131^I has been reported to be a critical affecting factor to the treatment effectiveness of ^131^I ablation. For example, Karam et al. (22) and Kim et al. (23) report that higher dose of ^131^I is associated with higher success rate of ^131^I ablation, but Kumar et al. (24) report that the success rate of therapy declines with increased ^131^I dose to DTC patients with lymph node and distant metastasis. We observe similar results showing that the efficacy of ^131^I ablation is reduced in the patients with metastasis. Specifically, compared to the group with no metastasis, the ablation success rate in patients with lymph node metastasis or distant metastasis is significantly lower. This is consistent with the finding of Zhang et al. that reports, when DTC patients are all given a fixed dose of 100 mCi, those with lymph node metastasis or distant metastasis have lower success rate of ^131^I ablation (18). Moreover, the success rate of thyroid remnant ablation is significantly lower in the intermediate- and high-risk groups than in the low-risk group. All of the above outcomes demonstrate that the success rate of the first ^131^I ablation declines when existence of lymph nodes or distant metastases from the primary tumor site. Therefore, metastasis is another determinant that could result in reduction of the efficacy of ^131^I ablation. The thyroid follicle epithelial cells of DTC can express Na(+)/I(-) symporter (NIS) and therefore retain the function and the ability of iodine uptake in varying extent (25). Radioiodine can also be taken up by the metastatic tissues, resulting in a reduction of the concentration of ^131^I in the thyroid tissue in DTC patients with metastases, and reduction of the efficacy of ^131^I ablation. In addition, some studies report that dedifferentiation and concomitant loss of thyroid–specific gene expression cause tumor cell resistance to ^131^I ablation, and this may constitute about 30–40% of patients with metastatic disease (26, 27).

Recently, Mäenpää et al. (28) and Ma et al. (29) report that administration of 30 mCi or 50 mCi is as effective as 100 mCi for ^131^I thyroid remnant ablation in low- to intermediate-risk DTC patients. However, other studies show that the success rate of the initial ^131^I thyroid remnant ablation in low-dose group (30–75 mCi) is lower than in high-dose group (100 mCi), suggesting that a higher success rate of ablation could be achieved by applying higher dose of ^131^I (28, 30, 31). In our hospital, DTC patients are routinely given a dose of 100 mCi and patients with suspected or confirmed microscopic residual lesions, lymph node metastasis, distant metastasis or unexplained serum Tg level increase are given a higher dose of ^131^I (100–200 mCi) according to the disease extent (e.g., extent of metastasis and the elevation of Tg level) (6).

Thyroglobulin is a glycoprotein, synthesized in thyroid cells or well-differentiated thyroid carcinoma cells. It is secreted together with thyroid hormones under the stimulus of TSH. Thyroglobulin is a critical parameter in predicting therapeutic response to ^131^I ablation and in monitoring recurrence or metastasis of thyroid carcinoma (32, 33). Many studies report that high sTg level before the first ^131^I ablation is associated with lower success rate of ^131^I ablation and sTg has an important predictive value for successful ^131^I ablation (13, 34–36). Tamilia et al. (37) and Zubair et al. (38) report that the cutoff sTg values for prediction of successful or unsuccessful thyroid remnant ablation are 6 and 18 ng/ml, respectively. In these two studies, the sensitivities of sTg to predict unsuccessful thyroid remnant ablation are 67 and 76.7%, respectively, and the specificities are 79 and 79.1%, respectively (37, 38). Our data also display that the success rate of the first ^131^I ablation is clearly lower in the group with higher pre-ablation sTg level. When sTg is >4.595 ng/ml, its sensitivity, specificity, positive predictive value and negative predictive value to predict thyroid remnant ablation failure are 95.51, 64.54, 58.22, and 96.52% respectively. These indicate that up to 96.52% of the patients with DTC may have successful ablation for the first time if the sTg is <4.595 ng/ml before ^131^I ablation. Therefore, the pre-ablation sTg measurement is an important predictor of the efficacy of ^131^I thyroid remnant ablation. The threshold value of sTg level in our study is similar to that of the Tamilia's report but is different from that of the Zubair's study. This discrepancy may be related to different research approaches, amount of the residual thyroid tissue, baseline TSH level, and detection method of thyroglobulin.

It is well known that the main factors affecting the thyroglobulin level include the amount of residual thyroid tissue or cancer lesion, TSH level, the time after the operation, and the thyroid hormone withdrawal time (39). These affecting factors can be closely related to each other. With the time extension post operation and thyroid hormone withdrawal, TSH level usually rises within a certain range, resulting in a parallel elevation of serum thyroglobulin. Therefore, to correct the influence of TSH on sTg level, we use sTg/TSH ratio and results show that when sTg/TSH is <0.046 mg/IU, up to 97.25% of DTC patients receiving ^131^I ablation is likely to succeed in ablation. Thus, sTg/TSH ratio is a reliable predictor of the efficacy of ^131^I thyroid remnant ablation and is helpful to the clinical decision-making in the radioiodine remnant ablation of DTC patients.

The 2015 ATA guidelines (8) propose clinical efficacy evaluation criteria, including ER, IDR, BIR, and SIR treatment responses as a dynamic risk assessment criterion after initial treatment. These are used to monitor disease outcomes timely and to adjust DTC risk stratification, subsequent follow-up, and treatment options. Our study also evaluate and compared the clinical efficacy of ^131^I ablation 6 months after treatment among no metastasis, no postoperative metastasis, postoperative lymph node metastasis, and distant metastasis groups, showing that the difference between the 4 groups is statistically significant. The ER rate of the non-metastasis group, the postoperative non-metastasis group, the postoperative lymph node metastasis group, and the distant metastasis group decreased gradually, while the SIR rate shows an upward trend. Further comparison of ER rates between the groups demonstrate that the non-metastasis group and the postoperative non-metastasis group are higher than those in the postoperative lymph nodes metastatic group or distant metastasis group and that the ER rates in postoperative lymph node metastasis group is higher than that of distant metastasis group. The correlation analysis shows that ER rate is negatively correlated with metastasis. The ER ratio of the successful group is significantly higher than that of the unsuccessful group. All of these results are consistent with previous studies (40, 41). On the one hand, it indicates that the clinical effect of DTC patients after ^131^I ablation depends on the presence and the severity of metastasis. On the other hand, the success rate of first ^131^I disinfection is an influencing factor of ER, that is, the higher the success rate of first ablation, the higher the possibility of ER and the better the prognosis. In view of the recurrence rate of ER patients is only 1–4% and the tumor related mortality risk is < 1% (42–44), the total thyroidectomy and the subsequent first ^131^I treatment should be optimized as far as possible so that the success rate of ^131^I ablation can be improved and more patients can enter the ER state as early as possible with the improved prognosis.

In summary, our study demonstrates that the success rate of ^131^I thyroid remnant ablation is high and that tumor size, pre-ablation sTg level, lymph node metastasis, and distant metastasis are the major factors affecting therapeutic success. Moreover, pre-ablation sTg and sTg/TSH ratio are reliable predictors of thyroid remnant ablation efficacy. When ^131^I ablation is performed in DTC patients, in order to optimize the ablation efficacy, to achieve the highest ER rate, and improve the prognosis, these factors should be taken into consideration in determining individualized ^131^I dosage and in anticipating the success potential of ^131^I ablation.

## Author Contributions

WZ developed the model, carried out the parameter estimations, and planned as well as performed the mass transfer experiments. CW wrote the main part of the manuscript and took part in the planning and execution of the fermentation experiments. HD took part in the development of the model, planned and carried out the main part of the fermentation experiments, analyzed the results, and assisted in the mass transfer experiments. PR helped analyze the results and assisted in the mass transfer experiments and wrote parts of the manuscript. XW and YW participated in the coordination of the study and reviewed the manuscript. All authors read and approved the final manuscript.

### Conflict of Interest Statement

The authors declare that the research was conducted in the absence of any commercial or financial relationships that could be construed as a potential conflict of interest.

## References

[1] Kim TY Kim WG Kim WB Shong YK. Current status and future perspectives in differentiated thyroid cancer. Endocrinol Metab. (2014) 29:217–25. 10.3803/EnM.2014.29.3.217 25309778 PMC4192824

[2] Yang X Liang J Li TJ Yang K Liang DQ Yu Z. Postoperative stimulated thyroglobulin level and recurrence risk stratification in differentiated thyroid cancer. Chin Med J. (2015) 128:1058–64. 10.4103/0366-6999.155086 25881600 PMC4832946

[3] Lee SJ Jung SL Kim BS Ahn KJ Choi HS Lim DJ. Radiofrequency ablation to treat loco-regional recurrence of well-differentiated thyroid carcinoma. Korean J Radiol. (2014) 15:817–26. 10.3348/kjr.2014.15.6.817 25469095 PMC4248639

[4] Shaha A. Prognostic factors in papillary thyroid carcinoma and implications of large nodal metastasis. Surgery (2004) 135:237–9. 10.1016/j.surg.2003.08.023 14739864

[5] Mazzaferri EL Kloos RT. Clinical review 128: current approaches to primary therapy for papillary and follicular thyroid cancer. J Clin Endocrinol Metab. (2001) 86:1447–63. 10.1210/jcem.86.4.7407 11297567

[6] Endocrinology Branch of Chinese Medical Association Guidelines for diagnosis and treatment of thyroid nodules and differentiated thyroid cancer. Chinese J Clin Oncol. (2012) 28:779–97.

[7] Cooper DS Doherty GM Haugen BR Kloos RT Lee SL Mandel SJ. Revised American Thyroid Association management guidelines for patients with thyroid nodules and differentiated thyroid cancer. Thyroid (2009) 19:1167–214. 10.1089/thy.2009.0110 19860577

[8] Haugen BR Alexander EK Bible KC Doherty GM Mandel SJ Nikiforov YE. 2015 American thyroid association management guidelines for adult patients with thyroid nodules and differentiated thyroid cancer: the American thyroid association guidelines task force on thyroid nodules and differentiated thyroid cancer. Thyroid (2016) 26:1–133. 10.1089/thy.2015.0020 26462967 PMC4739132

[9] NCCN NCCN Guidelines:Thyroid Carcinoma. Thyroid Cancer Version 3. The NCCN is the National Comprehensive Cancer Network (2011).

[10] Eustatiarutten CFA Corssmit EPM Biermasz NR Pereira AM Romijn JA Smit JW Survival and death causes in differentiated thyroid carcinoma. J Clin Endocrinol Metab. (2006) 91:313–9. 10.1210/jc.2005-1322 16263822

[11] Baudin E Schlumberger M. New therapeutic approaches for metastatic thyroid carcinoma. Lancet Oncol. (2007) 8:148–56. 10.1016/S1470-2045(07)70034-7 17267329

[12] Mazzaferri EL Jhiang SM. Long-term impact of initial surgical and medical therapy on papillary and follicular thyroid cancer. Am J Med. (1994) 97:418–28. 10.1016/0002-9343(94)90321-2 7977430

[13] Ha S Oh SW Kim YK Koo do H Jung YH Yi KH. Clinical outcome of remnant thyroid ablation with low dose radioiodine in korean patients with low to intermediate-risk thyroid cancer. J Korean Med Sci. (2015) 30:876–81 10.3346/jkms.2015.30.7.876 26130949 PMC4479940

[14] Verburg FA Lassmann M Mäder U Luster M Reiners C Hänscheid H. The absorbed dose to the blood is a better predictor of ablation success than the administered 131I activity in thyroid cancer patients. Eur J Nucl Med Mol Imaging (2011) 38:673–80. 10.1007/s00259-010-1689-5 21210115

[15] Liu Y Jin JH Liu JZ Li SJ Wu ZF Lu KY Efficacy and influencing factors of radioactive 131I for elimination of thyroid remnants following differentiated thyroid carcinoma resection. Chin Remedies Clin. (2013) 13: 556–8. 10.11655/zgywylc2013.05.003

[16] Shen Y Liu HC Hu YQ Tao J Sun WL Yu ZY Clinical efficacy of 131I treatment in 92 cases of differentiated thyroid cancer. Chin J Gen Pract. (2011) 9:1517–9. 10.16766/j.cnki.issn.1674-4152.2011.10.082

[17] Yuthana S Radioiodine remnant ablation in low-risk differentiated thyroid cancer. J Med Assoc Thai. (2013) 96:614–24. 10.1007/s12020-014-0523-4 23745318

[18] Zhang GZ Tan J Liu XH Meng ZW Influential factors on the effectiveness of 131I treatment on post-surgical differentiated thyroid cancer patients. Chin J Nucl Med. (2010) 30:259–63. 10.3760/cma.j.issn.0253-9780.2010.04.011

[19] Chen YH Ma YR Lin YS Kang ZS Li F Liu YM Comparison of different 131I doses for thyroid remnant ablation in patients with differentiated thyroid carcinoma. Chin J Nucl Med Mol Imaging (2012) 32:39–41. 10.3760/cma.j.issn.2095-2848.2012.01.012

[20] Fu HL Du XL Gu ZH Zou RJ Wu Z Wang H Analysis of influential factors for efficacy of 131I thyroid remnant ablation for differentiated thyroid carcinoma. J Shanghai Jiaotong Univ. (2010) 30:249–52.

[21] Zhao T Liang J Guo Z Li T Lin Y In patients with low- to intermediate-risk thyroid cancer, a preablative thyrotropin level of 30 μIU/mL is not adequate to achieve better response to 131i therapy. Clin Nucl Med. (2016) 41:454–8. 10.1097/RLU.0000000000001167 26914559

[22] Karam M Gianoukakis A Feustel PJ Cheema A Postal ES Cooper JAl. Influence of diagnostic and therapeutic doses on thyroid remnant ablation rates. Nucl Med Commun. (2003) 24:489–95. 10.1097/00006231-200305000-00002 12717064

[23] Kim EY Kim TY Kim WG Yim JH Han JM Ryu JS. Effects of different doses of radioactive iodine for remnant ablation on successful ablation and on long-term recurrences in patients with differentiated thyroid carcinoma. Nucl Med Commun. (2011) 32:954–9. 10.1097/MNM.0b013e32834956ec 21849927

[24] Kumar A Bal CS. Differentiated thyroid cancer. Indian J Pediatr. (2003) 70:707–13. 10.1007/BF02724312 14620185

[25] Mishra A Pal L Mishra SK. Distribution of Na + /I –, symporter in thyroid cancers in an iodine-deficient population: an immunohistochemical study. World J Surg. (2007) 31:1737–42. 10.1007/s00268-007-9156-6 17653791

[26] Schlumberger M Brose M Elisei R Leboulleux S Luster M Pitoia F. Definition and management of radioactive iodine-refractory differentiated thyroid cancer. Lancet Diabetes Endocrinol. (2014) 2:356–8. 10.1016/S2213-8587(13)70215-8 24795243

[27] Xing M. Molecular pathogenesis and mechanisms of thyroid cancer. Nat Rev Cancer (2013) 13:184–99. 10.1038/nrc3431 23429735 PMC3791171

[28] Mäenpää H O Heikkonen J Vaalavirta L Tenhunen M Joensuu H. Low vs. high radioiodine activity to ablate the thyroid after thyroidectomy for cancer: a randomized study. PLoS ONE (2008) 3:e1885. 10.1371/journal.pone.0001885 18382668 PMC2270902

[29] Ma C Feng F Wang S Fu H Wu S Ye Z. Chinese data of efficacy of low- and high-dose of iodine-131 for the ablation of thyroid remnant. Thyroid (2017) 27:832–7. 10.1089/thy.2015.0658 28401794

[30] Fallahi B Beiki D Takavar A Fard-Esfahani A Gilani KA Saghari M. Low versus high radioiodine dose in postoperative ablation of residual thyroid tissue in patients with differentiated thyroid carcinoma:, a large randomized clinical trial. Nucl Med Commun. (2012) 33:275–82. 10.1097/MNM.0b013e32834e306a 22124360

[31] Doi S A Woodhouse NJ. Ablation of the thyroid remnant and 131I dose in differentiated thyroid cancer. Clin Endocrinol. (2010) 52:765–73. 10.1046/j.1365-2265.2000.01014.x 10848882

[32] González C Aulinas A Colom C Tundidor D Mendoza L Corcoy R. Thyroglobulin as early prognostic marker to predict remission at 18–24 months in differentiated thyroid carcinoma. Clin Endocrinol. (2014) 80:301–6. 10.1111/cen.12282 23826916

[33] Ciappuccini R Hardouin J Heutte N Vaur D Quak E Rame JP. Stimulated thyroglobulin level at ablation in differentiated thyroid cancer: the impact of treatment preparation modalities and tumor burden. Eur J Endocrinol. (2014) 171:247–52. 10.1530/EJE-14-0192 24866576

[34] Li JH Wei ZX Zhao L Li Y Xu Q Analysis of the efficacy and influential factors of the first thyroid remnant ablation on differentiated thyroid carcinoma after operation. Shandong Med J. (2012) 52:13–5.

[35] Trevizam PGC Tagliarini JV Castilho EC de Alencar Marques M Kiy Y da Silva Mazeto GM. Thyroglobulin levels and thyroglobulin/thyrotropin ratio could predict the success of the ablative/therapeutic 131I in the differentiated thyroid cancers. Endocr Res. (2016) 42:1–7. 10.3109/07435800.2016.1173056 27144920

[36] Husseini MAE Implication of different clinical and pathological variables in patients with differentiated thyroid cancer on successful ablation for 3700 MBq 131I: a single Egyptian institutional experience over 14 years. Ann Nucl Med. (2016) 30:1–9. 10.1007/s12149-016-1084-9 27194041

[37] Tamilia M Al-Kahtani N Rochon L Hier MP Payne RJ Holcroft CA. Serum thyroglobulin predicts thyroid remnant ablation failure with 30 mCi iodine-131 treatment in patients with papillary thyroid carcinoma. Nucl Med Commun. (2011) 32:212–20. 10.1097/MNM.0b013e328341c802 21191314

[38] Zubair Hussain S Zaman MU Malik S Ram N Asghar A Rabbani U. Preablation stimulated thyroglobulin/TSH ratio as a predictor of successful i(131)remnant ablation in patients with differentiated thyroid cancer following total thyroidectomy. J Thyroid Res. (2014) 2014:610273. 10.1155/2014/610273 24987542 PMC4000651

[39] Pitoia F Abelleira E Cross G Thyroglobulin levels measured at the time of remnant ablation to predict response to treatment in differentiated thyroid cancer after thyroid hormone withdrawal or recombinant human TSH. Endocrine (2017) 55:200–8. 10.1007/s12020-016-1104-5 27655291

[40] Gao W Liang J Zhao T Li J Lin YS The impact of lymph node metastatic rate on clinical outcome following 131 I therapy in patients with papillary thyroid carcinoma. China Oncol. (2016) 26:67–72. 10.2217/fon.16.10

[41] Kim H Kim HI Kim SW Jung J Jeon MJ Kim WG. Prognosis of differentiated thyroid carcinoma with initial distant metastasis: a multicenter study in Korea. Endocrinol Metab. (2018) 33:287–95. 10.3803/EnM.2018.33.2.287 29947184 PMC6021319

[42] Han JM Kim WB Yim JH Kim WG Kim TY Ryu JS. Long-term clinical outcome of differentiated thyroid cancer patients with undetectable stimulated thyroglobulin level one year after initial treatment. Thyroid Official J Am Thyroid Assoc. (2012) 22:784–90. 10.1089/thy.2011.0322 22780573 PMC3407383

[43] Brassard M Borget I Edet-Sanson A Giraudet AL Mundler O Toubeau M. Long-term follow-up of patients with papillary and follicular thyroid cancer: a prospective study on 715 patients. J Clin Endocrinol Metab. (2011) 96:1352–9. 10.1210/jc.2010-2708 21389143

[44] Torlontano M Attard M Crocetti U Tumino S Bruno R Costante G. Follow-up of low risk patients with papillary thyroid cancer: role of neck ultrasonography in detecting lymph node metastases. J Clin Endocrinol Metab. (2004) 89:3402–7. 10.1210/jc.2003-031521 15240622

